# Development of Novel Murine BRAF^V600E^-Driven Papillary Thyroid Cancer Cell Lines for Modeling of Disease Progression and Preclinical Evaluation of Therapeutics

**DOI:** 10.3390/cancers15030879

**Published:** 2023-01-31

**Authors:** Grace Purvis Branigan, Victoria Casado-Medrano, Alison B. O’Neill, Julio C. Ricarte-Filho, Nicole Massoll, Madeleine Salwen, Zachary Spangler, Michele Scheerer, Edward K. Williamson, Andrew J. Bauer, Aime T. Franco

**Affiliations:** 1 Division of Endocrinology and Diabetes, Children’s Hospital of Philadelphia, Philadelphia, PA 19104, USA; 2 Department of Pathology, Winthrop P. Rockefeller Cancer Institute, University of Arkansas for Medical Sciences, Little Rock, AR 72205, USA; 3 Department of Pathology and Laboratory Medicine, Children’s Hospital of Philadelphia, Perelman School of Medicine, University of Pennsylvania, Philadelphia, PA 19104, USA; 4 Abramson Cancer Center, Perelman School of Medicine, University of Pennsylvania, Philadelphia, PA 19104, USA

**Keywords:** thyroid cancer, PTC, cell lines, mouse model, BRAF

## Abstract

**Simple Summary:**

Laboratory experimental models are essential for advancements in thyroid cancer translational research. In this study, we discuss the development and characterization of six cell-based models of BRAF^V600E^-driven papillary thyroid cancer that closely mimic the heterogeneous pathological progression of the disease seen in patients despite sharing a common driver mutation. We anticipate that these newly developed models will allow for the discovery of novel mechanisms that determine differences in disease progression among BRAF^V600E^-driven thyroid cancers and facilitate the testing of therapeutic interventions.

**Abstract:**

The Cancer Genome Atlas study in thyroid cancer exposed the genomic landscape of ~500 PTCs and revealed BRAF^V600E^-mutant tumors as having different prognosis, contrasting indolent cases and those with more invasive disease. Here, we describe the generation and characterization of six novel BRAF^V600E^-driven papillary thyroid cancer (PTC) cell lines established from a *Braf^V600E^*^+/−^/*Pten*^+/−^/*TPO-Cre* mouse model that spontaneously develop thyroid tumors. The novel cell lines were obtained from animals representing a range of developmental stages and both sexes, with the goal of establishing a heterogeneous panel of PTC cell lines sharing a common driver mutation. These cell lines recapitulate the genetics and diverse histopathological features of BRAF^V600E^-driven PTC, exhibiting differing degrees of growth, differentiation, and invasive potential that may help define mechanisms of pathogenesis underlying the heterogeneity present in the patient population. We demonstrate that these cell lines can be used for a variety of in vitro applications and can maintain the potential for in vivo transplantation into immunocompetent hosts. We believe that these novel cell lines will provide powerful tools for investigating the molecular basis of thyroid cancer progression and will lead to the development of more personalized diagnostic and treatment strategies for BRAF^V600E^-driven PTC.

## 1. Introduction

Thyroid cancer is the most common endocrine malignancy and the most frequently diagnosed cancer among adolescents and young adults between ages 15–29 [1,2,3]. Papillary thyroid cancer (PTC) is the most prevalent form of thyroid cancer and accounts for more than 90% of all thyroid cancer diagnoses (SEER Cancer Statistics Review, 1975–2017, National Cancer Institute). While well-differentiated (WD) PTC has an excellent prognosis, with a 5-year survival rate upwards of 90%, a small percentage of these tumors are believed to progress to poorly differentiated (PDTC) or anaplastic thyroid cancer (ATC). PDTC and ATC have a far worse prognosis, as they are often refractory to treatment and account for the majority of thyroid cancer-related mortality [4,5,6,7]. BRAF^V600E^ is the most common driver mutation of PTC in adults and the second most common driver mutation in children [8,9,10]. The BRAF^V600E^ mutation confers constitutive activation of the kinase, leading to sustained activation of the mitogen-activated protein kinase (MAPK) signaling pathway [11,12]. Clinical studies have associated the BRAF^V600E^ mutation with extrathyroidal extension, lymphatic and vascular invasion, local lymph node metastasis, higher clinical stage, persistent disease, reduced avidity for radioactive iodine, and lower survival rates in adults with PTC [10,11,12,13,14,15,16,17,18]. In contrast, the BRAF mutation has not been associated with the most invasive and metastatic disease in children with PTC; therefore, more data are needed to understand the natural history of BRAF-driven PTC in this population [8,19,20,21]. Notably, genetic alterations that trigger the activation of both the MAPK and phosphoinositide 3-kinase (PI3K)/Akt pathway become increasingly prevalent as disease progresses in adults. Moreover, it has been suggested that progression from PTC to ATC may be facilitated by mutations leading to the activation of the PI3K/Akt pathway co-occurring with BRAF mutations due to the increased mutation frequency in higher grade tumors [22,23,24,25].

Most PTCs are successfully managed with standard multimodal therapy. Patients undergo surgical tumor resection and lymph node dissection for localized disease, often followed by radioactive iodine (RAI) ablation, as adjuvant therapy in patients with high risk of recurrence or metastatic disease. Patients are then placed on thyroid hormone replacement therapy, with or without long-term thyroid hormone suppression, based on risk of recurrence. In the subset of patients with advanced and progressive RAI-refractory disease, oncogene-targeted therapies represent essential advancements in care and continue to be important areas of preclinical and clinical investigation in both adult and pediatric thyroid cancer.

It remains imperative to develop tumor-specific in vitro and in vivo preclinical models that recapitulate patient disease to prospectively evaluate treatments for efficacy and to decipher molecular mechanisms that confer therapeutic resistance. Although cell lines derived from human thyroid tumors provide a powerful tool to study novel therapies, research using human cells is limited to the tissue culture environment or xenograft models in immunocompromised hosts, which fails to recapitulate the interactions between tumor cells and the immune system in the host. Further, it was discovered that many previously reported thyroid tumor cell lines were either not unique or not actually of thyroid origin, further limiting the utility of some of these models [26]. In contrast, cell lines established from mouse models of thyroid cancer can be transplanted into immunocompetent syngeneic hosts, providing researchers with the ability to study drug responses in the physiologic tumor microenvironment. However, these cell lines do not always represent the heterogeneity observed in patient samples, and many of the previously reported lines are representative of advanced and anaplastic thyroid cancers [27,28]. We have previously reported the derivation of novel follicular thyroid cancer cell lines from Hras^G12V^-driven models of FTC [29] and sought here to develop cell lines derived from BRAF^V600E^-driven PTCs.

In this study, we detail the establishment of six independent cell lines from the *Braf^V600E^*^+/−^/*Pten*^+/−^/*TPO-Cre* mouse model of PTC. We have previously described a mouse model of PTC whereby thyroid-specific expression of *Braf^V600E^* induces development of thyroid tumors with 100% penetrance by five weeks of age [30]. Additionally, we have reported a mouse model of PTC utilizing the same thyroid-specific expression of *Braf^V600E^* and homozygous *Pten* inactivation in mice to achieve concomitant MAPK and PI3K/Akt pathway activation. These mice develop PTCs that rapidly progress to PDTC with 100% penetrance and lethality by weaning [31]. These models recapitulate the genetics and histopathological features of PTC as well as progress to a poorly differentiated state [30,31]. Here, we describe the establishment and characterization of six independent cell lines from murine thyroid tumors expressing *Braf^V600E^* and heterozygous *Pten*. Three cell lines were isolated from well differentiated PTC tumors (BB19, BB57, and BB342) and the other three cell lines were isolated from higher grade tumors with more invasive characteristics (BB1845, B1865, and B1866). This variability reproduces the clinical heterogeneity within the PTC patient population and, thus, allows for evaluation of characteristic features, in a subset of cell lines, that may be critical for disease progression. These cell lines represent novel and physiologically relevant research tools that can be used to evaluate therapeutic strategies and to illuminate factors that impact the progression of disease.

## 2. Materials and Methods

### 2.1. Derivation of Murine Thyroid Tumor Cell Lines and Wild-Type Thyrocyte Cultures

BB19, BB57, BB342, and BB1845 tumor cell lines were generated from thyroid tumors of *Braf^V600E^*^+/−^/*Pten*^+/−^/*TPO-Cre* mice of a pure 129/SvImJ genetic background (Jackson Laboratory mouse strain #:002448 here on referred to as 129SvJ). B1865 and B1866 tumor cell lines were established from thyroid tumors of *Braf^V600E^*^+/−^/*Pten*^+/−^/*TPO-Cre* mice of a mixed genetic background. Thyroid tumors or WT thyroids were dissected and minced. The pieces were then resuspended in Ham’s F12 medium (Corning, Glendale, AZ, USA) supplemented with 10% fetal bovine serum (FBS, Gibco), 2 mM L-Glutamine (Gibco), Penicillin/Streptomycin/Amphotericin B (Millipore-Sigma, Burlington, MA, USA), 10 µg/mL Piperacillin sodium salt (Millipore-Sigma, Burlington, MA, USA), and 10 µg/mL Ciprofloxacin Hydrochloride (Corning, Glendale, AZ, USA). Cells were then seeded into bind tissue culture flasks (Corning, Glendale, AZ, USA), and each flask was given an additional 500 μL of FBS. They were maintained at 37 °C in 5% CO_2_. After 24–48 h, the media were replaced, and the cells were maintained in 10% FBS F12 media supplemented with antibiotic for the next two weeks. WT thyrocytes were only cultured for at most one week before RNA was extracted to limit outgrowth of non-thyrocyte cells in culture. Then, antibiotic-free complete Ham’s 12 media was used. To rule out stromal cell contamination, all tumor cell lines were passaged at least 5 times after seeding and then genotyped using primers specific for Braf^V600E^ to check for complete recombination (Figure S2).

### 2.2. H&E and Trichrome Staining

Formalin fixed tissue was submitted to the Children’s Hospital of Philadelphia Pathology Core Laboratory for processing and embedding. All sectioning and staining were performed by the CHOP Pathology Core Laboratory. Trichrome histology slides were also scanned by the CHOP pathology core. H&E slides were evaluated by a pathologist blinded to experimental conditions.

### 2.3. RT-PCR Analysis

Total RNA from all cell lines was extracted using the Direct-zol RNA Miniprep (Zymo Research, Irvine, CA, USA). Equal amounts of RNA template were reverse transcribed using the Verso cDNA synthesis kit (Thermo Scientific, Waltham, MA, USA). The differential mRNA expressions of *Tg*, *Tshr*, *Nkx2-1* (*Ttf1*), *Pax8*, *Slc5a5* (*Nis*), and *B2m* were measured using pre-designed probes (Integrated DNA Technologies, Coralville, IA, USA) and Taqman master mix (Thermo Scientific, Waltham, MA, USA). A total of four µL of cDNA from tumor samples and independent passages of each cell line and WT thyroids were run in triplicate on the QuantStudio 3 (Thermo Fisher, Waltham, MA, USA). *B2m* was used as an internal control to normalize the expression levels of thyroid specific genes. Data analysis was based on the delta-delta Ct method.

### 2.4. Proliferation and Viability Assays

Cells were plated, 300 per well, in quadruplicate into 96-well plates. At designated time points, cell growth was assessed using the Cell Titer-Glo luminescent cell viability assay (Promega, Madison, WI, USA). Luminescence was determined using the Varioskan Lux (Thermo Scientific, Waltham, MA, USA). Day 0 represents 18 h after plating. Inhibitor studies were conducted using Dabrafenib methanesulfonate salt (D-5699, LC Laboratories, Woburn, MA, USA) and Trametinib (T-8123, LC Laboratories, Woburn, MA, USA) dissolved in DMSO. To assess the relative viability of cells in the presence of single inhibitors, Dabrafenib, Trametinib, or vehicle (DMSO) were added 18h after plating. Relative viability was assessed using the Cell Titer-Glo luminescent cell viability assay, and concentrations of Dabrafenib 10 nM and Trametinib 1 nM were selected for viability assays of the complete cell line panel.

### 2.5. Western Blot Analysis

To prepare total cellular protein lysates, 200,000 cells per well were seeded in six-well plates and allowed to attach for 24 h in complete medium. Cells were serum starved overnight before any specific treatment or stimulation. Then, cells were lysate in equal volumes of radioimmunoprecipitation (RIPA) buffer (50 mM Tris-HCl pH 7.4, 1% NP-40, 0.25% Na-deoxycholate, 150 mM NaCl, 1mM EDTA) supplemented with phosphatase and protease inhibitors (Thermo Scientific, Waltham, MA, USA) and 4× sample buffer (BioRad, Hercules, CA, USA) containing 200 mM DTT and boiled for 5 min. A small aliquot was taken after harvesting to measure protein concentration by BCA. To perform the SDS–polyacrylamide gel electrophoresis (PAGE), equal quantities of total cellular proteins from each sample were loaded into 10% acrylamide gels containing SDS-PAGE. Protein sizes were determined by comparison to a set of standards (Thermo Fisher, Waltham, MA, USA). To perform the immunoblot, proteins were transferred to a PVDF membrane pore size 0.45 μm (Millipore-Sigma, Burlington, MA, USA) after methanol activation. Primary antibodies (1:1000) specific to pERK (9101S), ERK (9102S), pAKT (9271S), AKT (4685S), PTEN (9188T), E-cadherin (14472s), Vimentin (5741S), and β-actin (3700S) (Cell Signaling Technologies (Danvers, MA, USA) were diluted in 5% bovine serum albumin (BSA) and 0.1% Tween-20 tris buffer (TBS-T) and incubated overnight at 4 °C. Specific binding of antibodies was detected with appropriate HRP-coupled secondary antibodies anti-rabbit (1:3000) or anti-mouse (1:5000) and visualized using enhanced chemiluminescence reagent (Thermo Fisher, Waltham, MA, USA). Protein expression levels were analyzed and compared by densitometry using iBright Analysis Software (Thermo Fisher, Waltham, MA, USA). Relative protein phosphorylation levels were obtained by normalizing the amount of phosphorylated protein to that of total protein. Original, uncropped Western blot membrane can be found in Figure S5. 

### 2.6. Organoids

Organoids were grown using previously described methods [32]. Briefly, to analyze the organoid formation rate in the presence of inhibitors, Dabrafenib, Trametinib, or vehicle (DMSO) were added to the media 18 h after plating. Organoids were grown for 10 days before imaging. Organoid size was measured using ImageJ particle analysis tool within each 3220 × 2415 micron area per image.

### 2.7. Migration Assay

Migration assays were performed in 24-well plates with cell culture inserts with 8.0 μm pores (Falcon, Chicago, IL, USA); 75,000 tumor cells were seeded in the top of each insert in serum free Hams F-12 media. Complete 10% FBS Hams F12 media was added to the bottom chamber of the plate. Cells were then incubated at 37 °C for 18 h. The cells were then fixed using 4% Paraformaldehyde, and the cells were removed from the top of the chamber using cotton tipped applicators. The underside of the chambers was then stained with crystal violet. Images were taken on the EVOS M7000 imaging system (Invitrogen by Thermo Fisher Scientific).

### 2.8. Imaging

Phase contrast imaging was performed using the EVOS M7000 imaging system (Invitrogen by Thermo Fisher Scientific). H&E histology slides were imaged using the EVOS M7000 imaging system (Invitrogen by Thermo Fisher Scientific). Trichrome histology slides were scanned by the CHOP pathology core and images captured using Image Scope. For differential interference contrast (DIC) imaging live cells were imaged with a Zeiss Axiovert 200M platform using a 40× EC Plan Neo Fluar, 1.3 N.A., or a 63× Plan Apochromat, 1.4 N.A. objective (Carl Zeiss, Jena, Germany) equipped with an Orca ER CCD camera (Hamamatsu, Bridgewater, NJ, USA). Images were captured using Slidebook 6 imaging software (Intelligent Imaging Innovations, Denver, CO, USA) using an Orca ER CCD camera (Hamamatsu).

### 2.9. In Vivo Tumorigenicity Assay

All animal experiments were performed at the Children’s Hospital of Philadelphia and approved by the IACUC. 5.0 × 10^5^ tumor cells (BB19, BB57, BB342, and BB1845) were pelleted by centrifugation, resuspended in 100 µL of a 1:1 mixture of Hams F-12 and Matrigel (Corning, Corning, NY, USA), and injected subcutaneously into the right hind flanks of 4–5-week-old male and female wild-type 129SvJ recipient mice. Tumor development was monitored weekly for 15 weeks. Mice were sacrificed due to tumor burden or loss of skin integrity as needed during that period or were collected finally at the end of the 15 weeks. Tumors were collected and fixed in 10% formalin for histological analysis.

### 2.10. Statistical Analysis

All data were analyzed using Prism 8 software (GraphPad, San Diego, CA, USA). Differences with *p*-values of ≤0.05 were considered statistically significant. All data comparing more than two groups were analyzed via ANOVA analysis with post hoc analysis (indicated in figure legends).

## 3. Results

### 3.1. Generation of Stable Tumor Cell Lines from Braf^V600E^ Murine Thyroid Tumors

**Figure 1:**
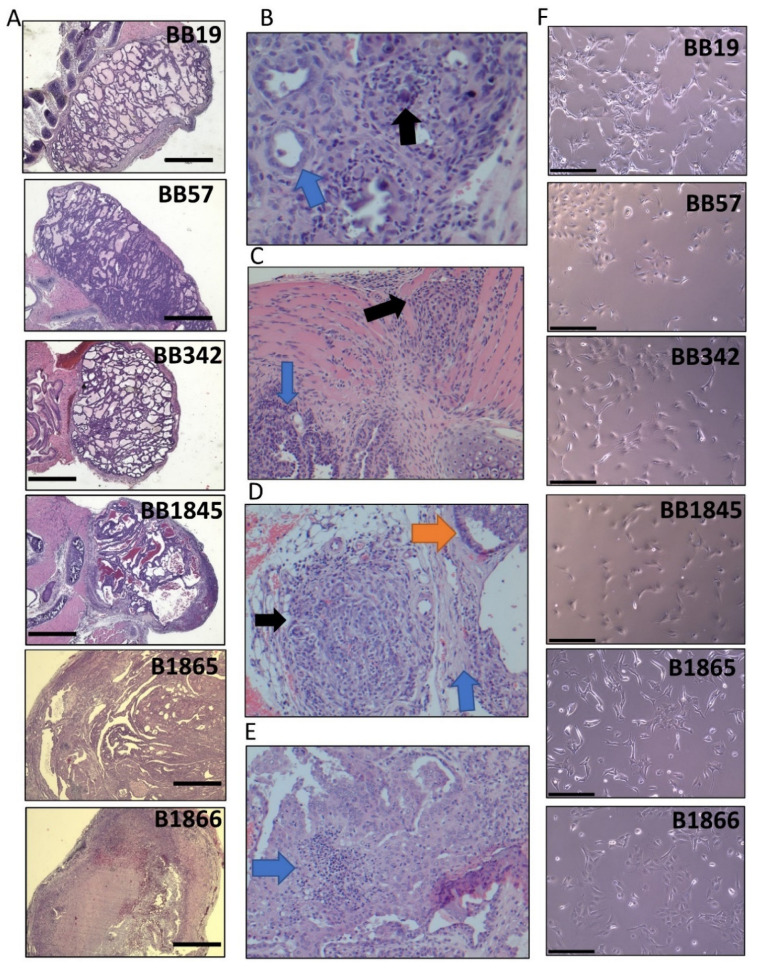
Histology and pathological features of Braf^V600E^ driven murine thyroid tumors and the morphology of stable tumor cell lines derived from them. (**A**) Hematoxylin and eosin (H&E) staining of de novo tumors for cell lines BB19, BB57, BB342, BB1845, B1865, and B1866. Scale bar in black is 750 µM. (**B**) High magnification of BB57 H&E showing papillary thyroid carcinoma with fine chromatin, nuclear grooves (blue arrow) and psammoma bodies (black arrow). (**C**) High magnification of BB57 H&E showing papillary thyroid carcinoma (blue arrow) with invasion into the muscle (black arrow). (**D**) High magnification of an extra-thyroidal nodule outside of the thyroid capsule (blue arrow), adjacent to intra-thyroidal tumor (orange arrow) in B1866. (**E**) High magnification of B1866 H&E showing moderately differentiated papillary thyroid carcinoma, which maintains papillae and grooves but has associated necrosis (blue arrow). (**F**) Representative phase contrast images of the individual cell lines in culture derived from the indicated tumors. Scale bar in black is 300 µM.

Papillary thyroid carcinomas developed in *Braf^V600E^*^+/−^/*Pten*^+/−^/*TPO-Cre* mice of a pure 129SvJ genetic background (cell lines BB19, BB57, BB342, and BB1845) or a mixed genetic background (cell lines B1865 and B1866). Tumors were collected from different sex animals and at a variety of time points from 3–24 weeks of age (Table 1). The majority of the tumor was digested to derive the cell lines with approximately 1/4 preserved for histopathologic analysis. Hematoxylin and eosin staining of the founding tumors for each cell line confirmed that the model exhibits PTC-like histologic features including papillae, nuclear grooves, psammoma bodies, fine chromatin (Figure 1A), and lymphocytic infiltration. BB57 exhibited classic PTC features such as fine chromatin, nuclear grooves, and psammoma bodies (Figure 1B), as well as invasion into the muscle (Figure 1C). In B1866, extra-capsular tumor extension was observed in association with the identified intra-thyroidal tumor (Figure 1D). Additionally, this tumor exhibits moderately differentiated PTC histology that maintains papillae and grooves but displays localized areas of necrosis (Figure 1E). Six independent cell lines were established from these six founding tumors, sharing the same genotype but derived from animals that varied in age and sex (Table 1). The lungs from the six founding mice were each examined for gross metastasis at time of dissection. No gross metastasis was observed at time of dissection, but suspicious areas were noted in the lungs of BB1845. Not metastasis could be confirmed by microscopic evaluation. Despite arising from tumors with the same driver mutation, the cells show heterogeneous morphologies among the six independent cell lines (Figure 1F). Heterogeneity in morphology and cell size is also observed within each cell line.

*Braf^V600E^*^+/−^/*Pten*^+/−^/*TPO-Cre* mice undergo *Braf* recombination specific to thyroid epithelium via the action of *TPO-Cre*. To confirm the presence of *Braf^V600E^* recombination in established cell lines, PCR analysis was performed using primers specific for *Braf* WT, *Braf^V600E^* recombined, and *Braf^V600E^* unrecombined alleles. As expected, each cell line exhibited one *Braf* WT band and one *Braf^V600E^* recombined band (Figure S1). In comparison, DNA isolated from non-target tissues from the tumor-bearing animals from which each the cell line was derived exhibited one *Braf* WT band and one *Braf^V600E^* unrecombined band, confirming that *Braf^V600E^* recombination was limited to thyroid epithelial cells. The absence of a *Braf^V600E^* unrecombined band in the six cell lines derived from these tumors confirms that the cell lines contained only thyroid epithelial cells, despite the morphologic heterogeneity of the cell lines.

### 3.2. Expression of Thyroid-Specific Genes, Proliferation Rate, and Activation of MAPK and PI3K Signaling Pathways in Novel Braf^V600E^ Murine Cell Lines

**Figure 2:**
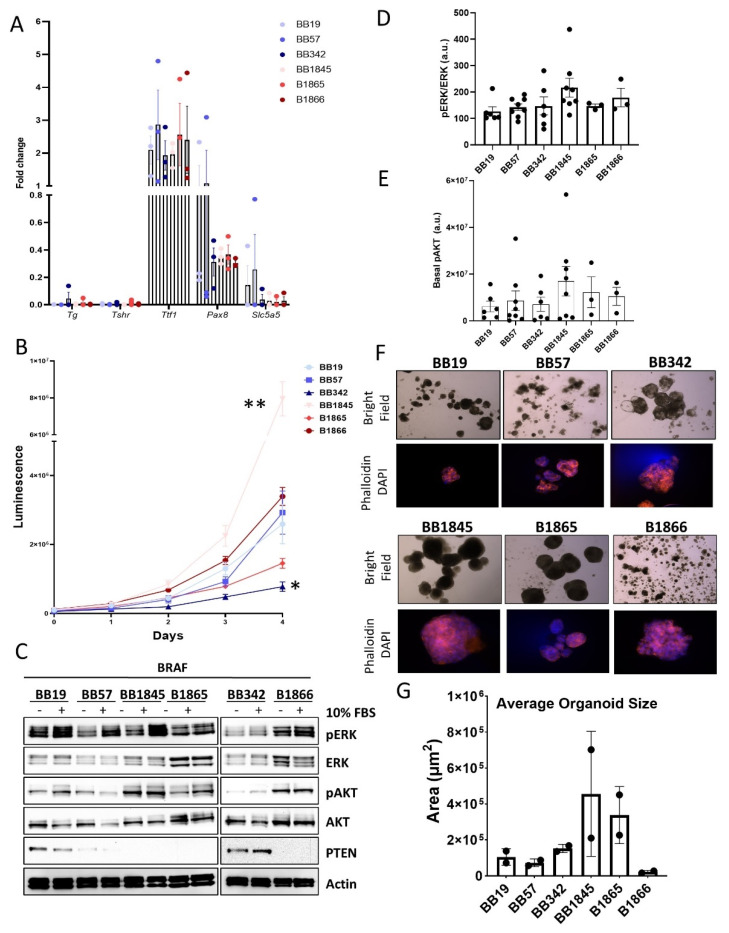
Expression of thyroid-specific genes, proliferation rate, and activation of MAPK and PI3K pathway signaling in novel BRAF^V600E^ murine cell lines. (**A**) Real-time qPCR analysis of the expression levels of thyroid-specific genes in primary wild-type (WT) thyrocytes and thyroid tumor cell lines in culture. Each sample was analyzed in triplicate, graph represents *n* = 3. Fold change is indicated on the y-axis. (**B**) Growth curves quantitating the growth of the cell lines in 2D cultures using Cell-Titer Glo cell viability assay. Day 0 represents 18 hours after plating. Each cell line was plated in quadruplicate, graph represents *n* = 3, * *p* > 0.05 compared to BB19, ** *p* > 0.002 compared to BB19. (**C**) Western Blot analysis characterizing differential activation of the MAPK and PI3K pathway between cell lines and differential levels of Pten. (**D**) Quantitation of ratio of pERK to total ERK levels in each cell line following 10 min 10% FBS simulation after overnight growth factor withdrawal. *n* = minimum of 3 independent replicates. (**E**) Quantitation of basal pAKT levels in the 6 cell lines. *n* = minimum of 3 independent replicates. (**F**) Representative images of organoid cultures from each cell line. Top panel represents bright field images imaged at 4× magnification, bottom panel is immunofluorescence images with Phalloidin in pink and DAPI (Nuclei) in blue, imaged at 40× magnification. (**G**) Quantitation of average organoid size from each cell line. Average sizes from two independent replicates.

The thyroid epithelial lineage and differentiation status of the cell lines was determined by measuring the mRNA levels of thyroid-specific genes including *Tg, Tshr*, *Nkx2-1* (*Ttf1*), *Pax8*, and *Slc5a5* (*Sodium Iodide symporter*: *Nis*) (Figure 2A). *Pax8* and *Ttf1* are transcription factors crucial to thyroid organogenesis, and their absence results in varying degrees of thyroid dysgenesis [33,34]. The expression of *Ttf1* was similar among the six cell lines and was higher when compared to the expression in WT thyrocytes. *Pax8* expression was lower than in WT thyrocytes in most cell lines, except BB19 an BB57, which had expression similar to that observed in WT thyrocytes. *Pax8* and *Ttf1* regulate the expression of the thyroid-specific genes *Tg*, *Tshr*, and *Slc5a5* (Nis) [34]. *Tg* and *Tshr* expression levels were similar among the six cell lines but were very low compared to expression in WT thyrocytes. In contrast, *Nis* expression was variable between the six cell lines but was consistently much lower compared to WT thyrocytes. Like thyroid transcription factors, loss of *Tg*, *Tshr*, or *Nis* expression has been associated with PTC disease progression [35,36,37,38]. Loss of *Nis* expression is particularly relevant clinically as it is associated with disease refractory to RAI ablation therapy [39,40,41].

We next sought to evaluate the proliferation rates of the cell lines in 2D culture using the Cell Titer Glo luminescence-based viability assay (Figure 2B). The Cell Titer Glo method is based on the quantitation of ATP to determine the number of metabolically active cells. Most of the cell lines had a lag-phase of approximately 48 h. At day three, BB1845, B1866, and BB19 appeared to enter logarithmic growth, whereas BB57 entered logarithmic growth at day 4. B1865 and BB342 did not enter logarithmic growth phases within 4 days. Maximal differences were observed at day 4 clearly showing heterogeneity in proliferation rates among the six cell lines. BB1845 was the most proliferative cell line, while B1865 and BB342 had the longest lag phases.

Next, Western blot was used to assess the MAPK and PI3K pathway activation in response to acute FBS stimulation and to quantify their basal levels after growth factor deprivation (Figure 2C). The cell lines displayed heterogeneous basal levels of ERK, pERK, Akt, and pAKT, with little increase in pERK and pAKT in response to FBS stimulation (Figure 2C,D). As described previously, all of the cell lines were derived from mice harboring tumors with thyrocyte-specific heterozygous deletion of *Pten*. To determine whether levels of PTEN protein expression correlated with basal pAKT levels, Western blot analysis was performed for PTEN. Consistent with their elevated basal pAKT expression, BB57, BB1845, B1865, and B1866 all showed reduced or no expression of PTEN protein (Figure 2C,E). PCR analysis using primers specific for WT *Pten*, un-recombined mutant *Pten*, and recombined *Pten*, subsequently showed that for cell lines BB1845, B1865, and B1866, the WT copy of *Pten* had been lost (Figure S2). However, the WT copy of *Pten* was retained in BB19, BB57, and BB342 suggesting that the decreased PTEN expression seen in BB57 is due to another mechanism.

Historically, the most common method of in vitro cell culture has been the growth of cells in a two-dimensional monolayer on cell culture plastic. However, three-dimensional (3D) models of tumor growth may be more relevant as they better recapitulate the natural environment and structure of solid tumors. To characterize the growth of these novel cell lines in more physiologic conditions, we used an organoid forming assay (Figure 2D). BB342, BB1845, and B1865 formed large organoids, many with multiple lobes or protrusions from a central mass (Figure 2F,G). In contrast, BB19, BB57, and B1866 formed more numerous organoids, but they remained small in size (Figure 2F,G).

### 3.3. Evaluation of EMT Properties of Braf^V600E^ Cell Lines

**Figure 3:**
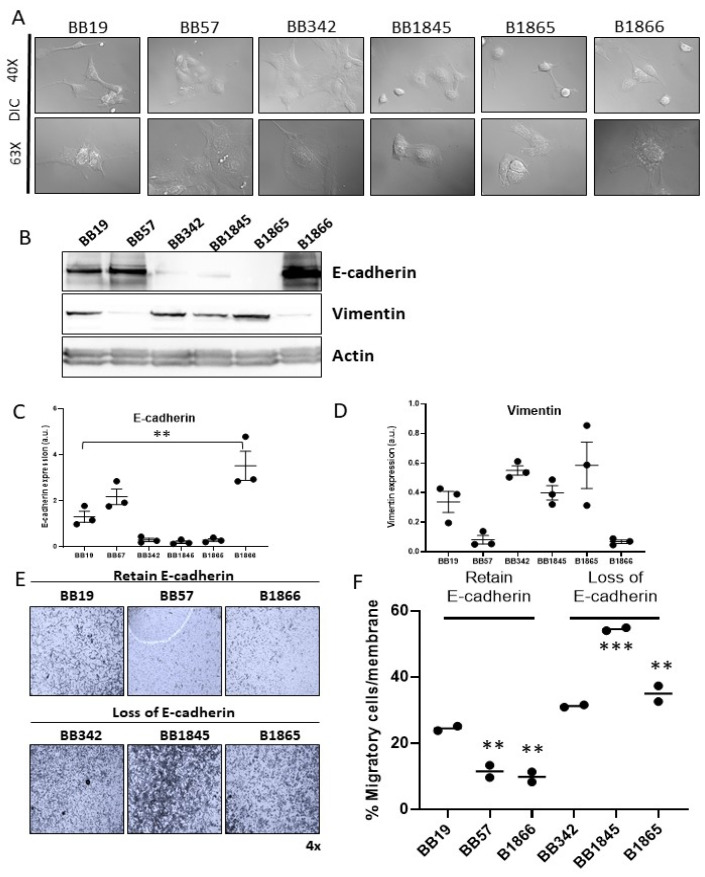
Characterization of epithelial and mesenchymal features of Braf^V600E^ cell lines. (**A**) Representative differential interference contrast (DIC) images of each cell line. Top: DIC imaging of live cells at 40×. Bottom: DIC imaging of live cells at 63×. (**B**) Western blot analysis of levels of epithelial (E cadherin) and mesenchymal (Vimentin) proteins. (**C**) Quantitation of E cadherin expression in each of the six cell lines N = 3, ** *p* < 0.002 compared to levels in BB19. (**D**) Quantitation of vimentin expression in each of the six cell lines N = 3. No Statistically different comparison compared to levels in BB19. (**E**) Representative images of cells that have migrated through the membrane of a trans-well, imaged at 4× magnification. (**F**) Quantification of the cells that have migrated through a trans-well. Percent of the membrane area covered by cells that have migrated through the filter is indicated on the y-axis. N = 2 independent experiments. ** *p* < 0.02 compared to BB19, *** *p* < 0.0001 compared to BB19.

Heterogeneity in cell morphology can be observed among the six cell lines (Figure 1F and Figure 3A). Despite their shared thyroid epithelial cell origin, each display different morphology and adhesion to neighboring cells. Tumor cells of epithelial origin are known to be able to undergo epithelial to mesenchymal transition (EMT) to aid in their escape from the primary tumor site and travel to a distant metastatic site [42]. The process of EMT alters cell morphology and transcriptional output from the tightly adherent epithelial cells expressing epithelial markers such as E-cadherin and cytokeratin to a fusiform appearance with expression of vimentin and fibronectin, typical of mesenchymal cells [42]. As the founding tumors of our novel cell line panel displayed varying degrees of invasion, we sought to determine whether proteins associated with EMT were dysregulated in the cell lines. Western blot analysis showed that BB19, BB57, and B1866 all maintained E-cadherin expression and had little vimentin expression. In contrast, cell lines BB342, BB1845, and B1865 had low to absent expression of E-cadherin and expressed high levels of vimentin (Figure 3B–D). To determine whether loss of E-cadherin correlated with migratory capacity of the cells, a modified Boyden-chamber migration assay was performed. A greater proportion of cells migrated through the transwell in the three cell lines lacking E-cadherin protein expression (BB342, BB1845, and B1865) (Figure 3E,F). In contrast, cells lines that maintained E-cadherin protein expression (BB19, BB57, and B1866) had reduced migratory capacity (Figure 3D,E).

### 3.4. Growth Suppressive Effect of Pathway-Specific Inhibitors in Braf^V600E^-Driven Thyroid Tumor Cell Lines in 2D Monolayer and 3D Organoid Cultures

**Figure 4:**
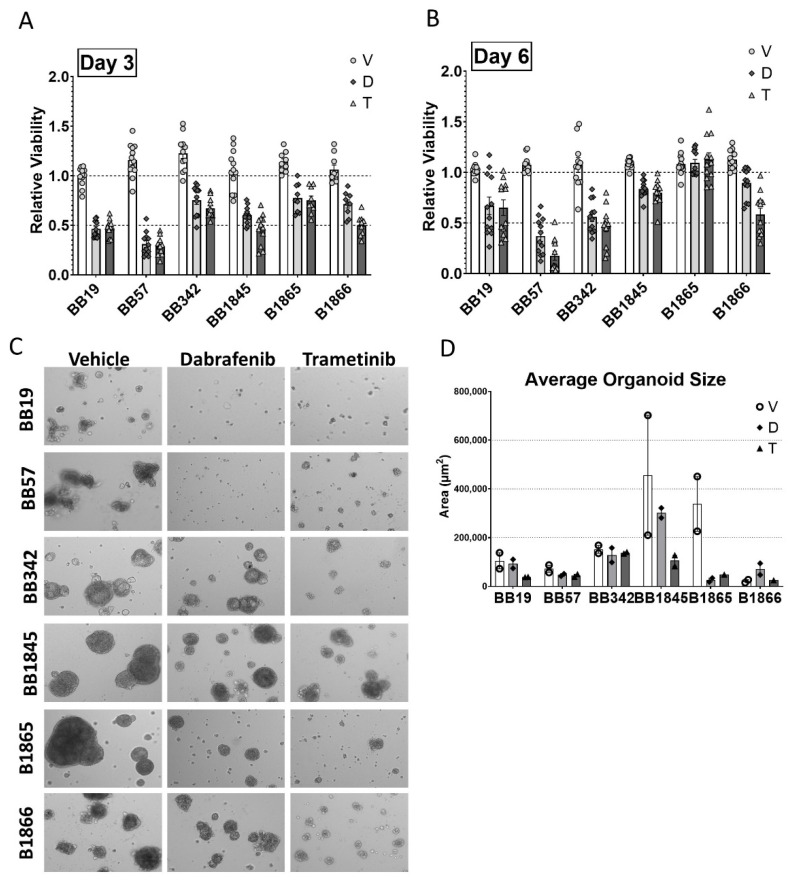
Growth-suppressive effects of targeted BRAF and MEK1/2 inhibition in *Braf^V600E+/−^/Pten^+/−^/TPO-Cre* cell lines in 2D monolayer and 3D organoid cultures. (**A**) Summary plot showing viability of novel cell lines in 2D culture following treatment with the BRAF inhibitor Dabrafenib (D), MEK1/2 inhibitor Trametinib (T) or DMSO vehicle (V) compared to control cells grown in standard culture medium. Cells were treated with selected concentrations of each inhibitor or vehicle for 3 days prior to analysis using CellTiter Glo Luminescent Cell Viability Assay. N = 3 independent experiments in triplicate. (**B**) Summary plot showing viability of tumor cell lines treated with inhibitors as in (**A**) for 6 days prior to analysis using CellTiter Glo Luminescent Cell Viability Assay. (**C**) Representative images of novel cell lines grown in 3D organoid culture and treated with the BRAF inhibitor Dabrafenib, MEK1/2 inhibitor Trametinib or DMSO vehicle for 10 days. Images were captured at 4× magnification. (**D**) Quantitation of average organoid area of novel cell lines treated as in (**C**).

We next evaluated the efficacy of MAPK specific inhibitors on the growth of these novel cell lines. Each cell line was treated with a variety of concentrations of either Dabrafenib (BRAF inhibitor) or Trametinib (MEK1/2 inhibitor) for 3 and 6 days (Figure S3). Both of these inhibitors are being utilized in clinical trials in thyroid cancer [43] and have been utilized in the pediatric setting [44]. In all cell lines, the inhibition of BRAF or MEK1/2 alone resulted in a significant reduction of growth at 3 days (Figure 4A). There was similar efficacy between BRAF and MEK1/2 inhibition in BB19, BB57, BB342, and B1865, whereas BB1845 and B1866 seemed to show slightly more resistance to BRAF inhibition alone at 3 days (Figure 4A). By six days of treatment, BB1845, B1865, and B1866 showed reduced or no significant growth inhibition with the BRAF inhibitor, and BB1845 and B1865 similarly showed reduced or no growth inhibition with the MEK1/2 inhibitor (Figure 4B). Notably, the cell lines showing reduced response to pathway-specific inhibition at 6 days correlated with those derived from higher grade founding tumors and with loss of PTEN expression (Figure S3).

To evaluate the growth-inhibiting effects of these drugs in a more physiologic environment, we established 3D organoid cultures of each cell line and assessed the size of the organoids following treatment with Dabrafenib, Trametinib, or DMSO control for 10 days (Figure 4C). Average organoid size and total organoid area measurements recapitulated expected growth inhibition following inhibitor treatment, as demonstrated in monolayer cultures (Figure 4D,E). Interestingly, in organoid cultures, the duration of response seemed to be maintained. By 6 days post treatment, most of the cell lines showed reduced response to inhibition in 2D, whereas inhibition of spheroid size as maintained up to 10 days in 3D culture.

### 3.5. Development of a Syngeneic Subcutaneous Tumor Model

The subcutaneous injection of tumor cells into murine hosts has been proven to be a valuable and convenient method for studying tumorigenesis. The very rapid growth and progression of *Braf^V600E^*^+/−^/*Pten*^+/−^/*TPO-Cre* de novo tumors in the mouse thyroid gland precludes many in situ experiments. Therefore, the development of a subcutaneous model in immune competent animals would enable temporal models to study disease progression and to potentially serve as a future model for investigating targeted therapeutic interventions. The strengths of the subcutaneous tumor models coupled with the rapidly penetrant phenotype of our de novo *Braf^V600E^*^+/−^/*Pten*^+/−^/*TPO-Cre* murine model [30,31] led us to develop a syngeneic tumor model of PTC. We evaluated the tumorigenic capacity of the pure 129SvJ genetic background cell lines (BB19, BB57, BB342, and BB1845) in immunocompetent hosts. Wild-type 129SvJ mice were injected with tumor cells subcutaneously into the right hind flank at 4–5 weeks of age. The mice were monitored three times a week for 15 weeks to track the development of tumors.

**Figure 5:**
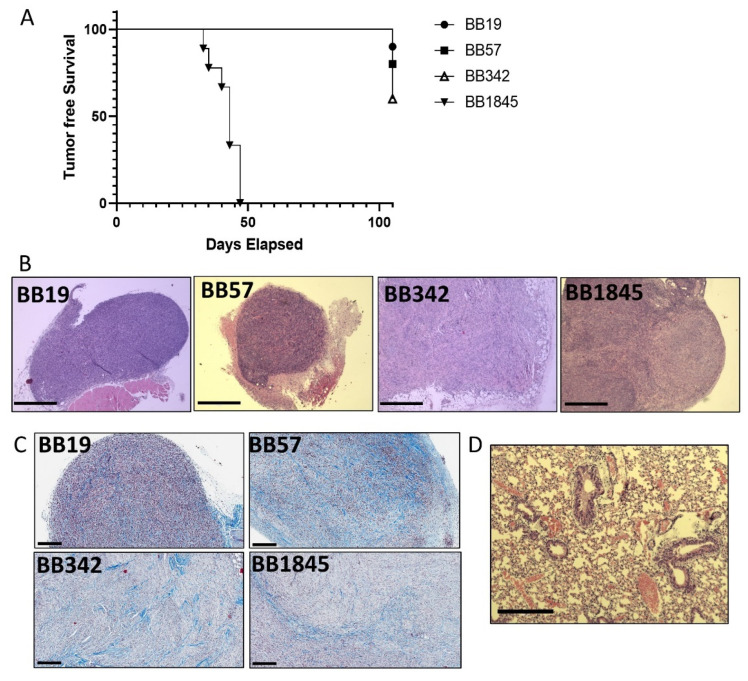
Cell lines develop syngeneic subcutaneous tumors. (**A**) Kaplan Meyer plot of tumor free survival of animals subcutaneously injected with Braf-driven tumor cell lines. (Note only 4 cell lines were on congenic 129SvJ background and therefore able to be syngeneically injected into WT 129SvJ recipients). (**B**) Representative H&E and images of subcutaneous tumors. Black scale bars are 750 µM. (**C**) Representative Trichrome images of subcutaneous tumors. Collagen is blue, nuclei are black, and muscle tissue erythrocytes and cytoplasm are red to pink. Black scale bar is 200 µM. (**D**) Representative H&E image of a lung metastasis. Black scale bar is 300 µM.

Similar to our previous studies with Hras^G12V^-driven tumor cells, variable tumor penetrance was observed between each of the cell lines. BB19 and BB57 were the least penetrant, whereas BB342 had an intermediate penetrance and BB184-injected mice developed tumors with 100% penetrance (Table 2). The latency and progression of tumors was much slower in BB19, BB57, and BB342 where all animals survived to 15 weeks post injection. By contrast, tumors developed very rapidly in mice injected with BB1845 whereby all mice had to be collected before the end point at 15 weeks (Table 2 and Figure 5A).

We sought to determine whether subcutaneous tumors were able to recruit a tumor microenvironment similar to the observed in *Braf^V600E^*^+/−^/*Pten*^+/−^/*TPO-Cre* de novo tumors in the thyroid [31]. To corroborate that characteristic, we analyzed the recruitment of activated fibroblasts that secrete a collagen-rich stroma (Figure S4). Trichrome staining of tumor sections confirmed a collagen-rich microenvironment.

We also sought to determine whether tumors developed in these mice would also undergo spontaneous metastasis to distant sites. All mice were evaluated for distant metastases at sacrifice. Lung metastases were observed in one out of four tumor-bearing mice for the cell line BB342 and in two out of nine tumor-bearing mice for the cell line BB1845 (Table 2 and Figure 5D).

## 4. Discussion

Cancer cell lines harboring clinically relevant mutations derived from genetically engineered mouse models are invaluable tools for in vitro and in vivo preclinical research to drive advancements in our understanding of disease mechanisms and treatment approaches in thyroid cancer. Here, we sought to generate cell lines that would recapitulate the heterogeneity of PTC and that would allow us to examine factors that contribute to disease progression. PTC is the most prevalent form of thyroid cancer, and incidence is steadily increasing [3]. *BRAF^V600E^* is the most common driver mutation in adult PTC and the second most common mutation in pediatric PTC [5,9,10,11,12,45]. Genetic alterations leading to activation in both the MAPK and PI3K/Akt pathway commonly occur in PTC and become increasingly more prevalent as disease progresses to PDTC and ATC [22,23,24,25]. According with these genetic trends, we have previously shown that thyroid-specific expression of *Braf^V600E^* alone or combined with *Pten* inactivation *(Braf^V600E^*^+/−^/*Pten*^−/−^/*TPO-Cre)* leads to the development of PTC in mice [30,31]. In addition to harboring clinically relevant mutations, these models also reproduced similar histopathologic PTC features and stages of progression to PDTC.

In this study, we described six novel independent murine PTC cell lines that were derived from *Braf^V600E^*^+/−^/*Pten*^+/−^/*TPO-Cre* mouse thyroid tumors. These cell lines share the same initiating driver mutations but represent a heterogenous population regarding sex, age, and histologic differentiation status. Additionally, we shown that these cell lines can be used for a variety of in vitro applications including mechanistic studies of targeted inhibitors and formation of 3D organoid cultures, while also demonstrating successful transplantation into syngeneic hosts for in vivo studies. Altogether, our results demonstrate heterogeneity within these cell lines that recapitulate differences observed clinically in the patient population. This novel cell line panel provides an innovative research tool to identify differences among tumor cells with heterogenous responses to treatment and/or invasive behavior despite sharing the same initiating driver mutations. Previous studies have indicated that progression from PTC to PDTC or ATC may be facilitated by mutations leading to the activation of the PI3K/Akt pathway co-occurring with *BRAF* mutations [22,23,24,25]. Interestingly, cell lines BB1845, B1865, and B1866, which arose from higher grade tumors, have lost their wild-type copy of *Pten* (Figure S3). These cell lines also demonstrated decreased susceptibility to MAPK pathway inhibition compared with the three cell lines derived from tumors with well-differentiated histology. These findings support the utility of our new cell line panel in studying mechanisms of disease advancement and elucidating prognostic features that may allow the early identification of more indolent versus more invasive tumors despite a common oncogene mutation.

Epithelial-to-mesenchymal transition is associated with loss of E-cadherin expression resulting in loss of cellular adhesion and polarity that leads to an invasive cell phenotype with increased metastatic potential [42,46,47].Previous studies have described EMT in the setting of PTC characterized by over-expression of vimentin in addition to increased tumor invasion and lymph node metastases [48]. We demonstrated that our cell lines showing loss of E-cadherin expression and gain of vimentin expression had greater migratory properties when evaluated using a trans-well migration assay (Figure 5C,D). Interestingly, the thyroid cell lines maintaining the most robust E-cadherin expression formed the smallest yet most frequent organoids. This is in contrast to studies in prostate organoid cultures whereby loss of E-cadherin results in reduced organoid size and disorganized organoid formation [49]. Our in vivo findings further support that loss of E-cadherin expression and increased vimentin expression is advantageous to hematogenous metastasis, indicated by the lung metastasis observed in animals injected with BB342 and BB1845. These novel cell lines will be foundational to future studies to investigate the differential invasiveness and metastatic potential of PTC, and the role of EMT proteins in organoid formation.

The current understanding of EMT in thyroid carcinomas suggests that EMT is implicated in the progression from well-differentiated thyroid cancers to PDTC and ATC [42,50,51,52]. However, our data have mixed results to this end. One of our cell lines with well-differentiated histology, BB342, displayed a mesenchymal-like phenotype characterized by loss of E-cadherin expression, gain of vimentin expression, and increased migratory properties as well as increased metastatic potential in vivo. These findings, in the setting of the well-differentiated histology of the founding tumor, suggest that EMT can occur early in tumorigenesis and may lead to early tumor progression. Interestingly, one of our cell lines that originated from a higher-grade tumor, B1866, was shown to have maintained its epithelial characteristics despite coming from an advanced stage tumor. Together, these results highlight the complex and multifactorial nature of primary tumor progression and the closely related but distinct processes of invasion and metastasis. Furthermore, our findings demonstrate that this novel cell line panel provides a platform that will allow researchers to investigate the mechanisms by which these cell lines developed heterogeneous tumor cell phenotypes despite sharing a common initiating tumorigenic event. Using this model, investigators may gain crucial insights into early prognostic indicators of tumor progression that may supplement the identification of the underlying oncogene mutation and allow for advanced precision in therapeutic strategies for patients with newly diagnosed, recurrent, or RAI-refractory PTC.

In this study, we sought to validate a syngeneic subcutaneous tumor model. This provides a readily accessible method of exploring tumorigenesis that recapitulates the complex interactions between tumor cells, the immune system, and physiologic components of the tumor microenvironment. Previous studies have shown that immune cell recruitment, particularly macrophages, plays a role in thyroid cancer pathogenesis [53,54,55]. Further, immune modulating therapy is now being tested in clinical trials for advance thyroid cancer. These novel models will enable comprehensive investigation of thyroid oncogenesis and response to therapy, including immune modulatory therapies. We demonstrate that the derived cell lines display different latencies and penetrance of tumor formation, and differential capacity to spontaneously metastasize to the lungs. One limitation of our in vivo findings using this model is that the histology observed in the subcutaneous tumors is characteristic of advanced disease for all the cell lines evaluated, despite the heterogeneous differentiation states of the founding tumor for each cell line. However, we anticipate that these cell lines could be used in more advanced in vivo modeling systems, including orthotopic implantation directly into the thyroid of wild-type recipients. It is possible that under those circumstances the resulting tumors could have histological features that are more representative of well-differentiated PTC.

Additionally, this model could be used to evaluate the role of age at onset of disease in PTC development and progression by injecting hosts of different age equivalents to compare penetrance, disease progression, and metastasis [56,57,58,59,60,61]. While the *BRAF^V600E^* mutation is very common in adult PTC and moderately common in pediatric PTC, the mechanisms behind the disparity in clinical outcomes between these two populations is not well-understood. We believe that these cell lines model the heterogeneity seen in the population of patients with BRAF-driven PTC and provide a novel platform to investigate features critical to PTC disease progression and response to therapies. These tools provide a valuable platform for more reductionist and mechanistic approaches to investigate Braf-driven disease progression.

## 5. Conclusions

The establishment of six independent cell lines modeling BRAF^V600E^-driven papillary thyroid cancer represents an advancement in the development of much-needed validated preclinical models for thyroid cancer research. We anticipate that these cell lines will provide a powerful platform for in vitro and in vivo studies that will lead to the development of precision diagnostic and treatment strategies for patients with newly diagnosed, advanced, or treatment-refractory thyroid cancer. Ongoing work in the laboratory is seeking to identify molecular mechanisms that drive heterogeneity observed in morphology and responses to therapy of these cell lines, despite sharing identical driver mutations.

## Figures and Tables

**Table 1:** Demographic information of the mice from which tumor cell lines were derived.

Cell Line	Genotype	Sex	Age (Weeks) at Collection
BB19	*BrafV600E^Het^*/*Pten^Het^*/*TPO-Cre+*	Female	6
BB57	*BrafV600E^Het^*/*Pten^Het^*/*TPO-Cre+*	Male	4
BB342	*BrafV600E^Het^*/*Pten^Het^*/*TPO-Cre+*	Male	3
BB1845	*BrafV600E^Het^*/*Pten^Het^*/*TPO-Cre+*	Female	32
B1865	*BrafV600E^Het^*/*Pten^Het^*/*TPO-Cre+*	Male	24
B1866	*BrafV600E^Het^*/*Pten^Het^*/*TPO-Cre+*	Male	24

**Table 2:** Summary of tumor penetrance and lung metastasis of injected cell lines.

Cell Line	Number of Animals with Tumors	% Penetrance (/N)	Number of Animals with Lung Metastasis
BB19	1	10% (1/10)	0
BB57	2	20% (2/10)	0
BB342	4	40% (4/10)	1
BB1845	9	100% (9/9)	2

## Data Availability

Data supporting the findings of this study are contained within the article and Supplementary Materials or are available from the corresponding author (A.T.F.) upon reasonable request.

## Supplementary Materials

The following supporting information can be downloaded at: https://www.mdpi.com/article/10.3390/cancers15030879/s1, Figure S1: Trichrome of original de novo tumors; Figure S2: Braf Recombined DNA gels on cell lines and non-target tissues; Figure S3: Pten Recombined DNA gel on cell lines and non-target tissues and original Pten Genotyping gels for BB1845, B1865, and B1866; Figure S4: Dose-dependent growth-suppressive effects of targeted BRAF and MEK1/2 inhibition in *BrafV600E*^+/−^/*Pten*^+/−^/*TPO-Cre* cell lines; Figure S5. Original, uncropped Western blot membrane.
