# Supplementary material for: Development of Novel Murine BRAFV600E-Driven Papillary Thyroid Cancer Cell Lines for Modeling of Disease Progression and Preclinical Evaluation of Therapeutics
Source: Cancers (Basel). 2023 Jan 31;15(3):879. doi: 10.3390/cancers15030879 (PMC9913801; doi:10.3390/cancers15030879)
Supplement: Supplementary file 1 [file cancers-15-00879-s001.zip › cancers-1981262 -Figure S5.pdf]

a:

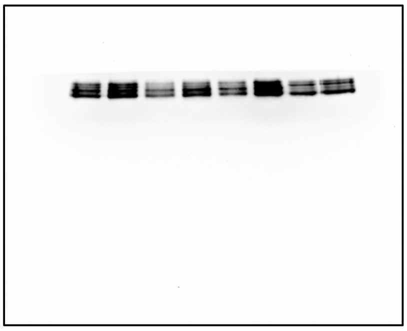

Lane 1_pERK_ BB19 -10% FBS
Lane 2_pERK_ BB19 +10% FBS
Lane 3_ pERK_ BB57 -10% FBS
Lane 4_ pERK_ BB57 +10% FBS
Lane 5_ pERK_ BB1845-10% FBS
Lane 6_ pERK_ BB1845 +10% FBS
Lane 7_ pERK_ BB1865 -10% FBS
Lane 8_ pERK_ BB1865 +10% FBS

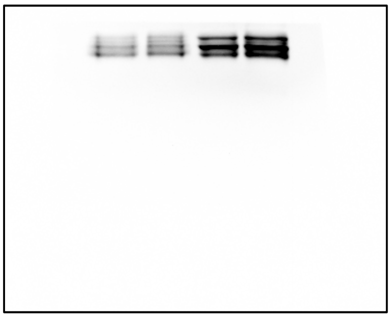

Lane 1_pERK_ BB342 -10% FBS
Lane 2_pERK_ BB342 +10% FBS
Lane 3_ pERK_ BB1866 -10% FBS
Lane 4_ pERK_ BB1866 +10% FBS

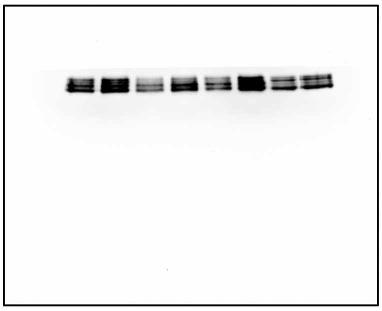

Lane 1_ERK_ BB19 -10% FBS
Lane 2_ERK_ BB19 +10% FBS
Lane 3_ ERK_ BB57 -10% FBS
Lane 4_ ERK_ BB57 +10% FBS
Lane 5_ ERK_ BB1845-10% FBS
Lane 6_ ERK_ BB1845 +10% FBS
Lane 7_ ERK_ BB1865 -10% FBS
Lane 8_ ERK_ BB1865 +10% FBS

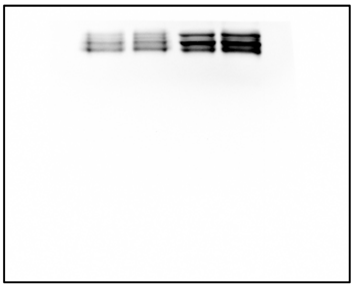

Lane 1_ERK_ BB342 -10% FBS
Lane 2_ERK_ BB342 +10% FBS
Lane 3_ ERK_ BB1866 -10% FBS
Lane 4_ ERK_ BB1866 +10% FBS

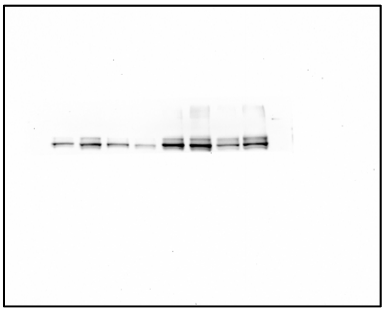

Lane 1_pAKT_ BB19 -10% FBS
Lane 2_pAKT_ BB19 +10% FBS
Lane 3_ pAKT _ BB57 -10% FBS
Lane 4_ pAKT _ BB57 +10% FBS
Lane 5_ pAKT _ BB1845-10% FBS
Lane 6_ pAKT _ BB1845 +10% FBS
Lane 7_ pAKT _ BB1865 -10% FBS
Lane 8_ pAKT_ BB1865 +10% FBS

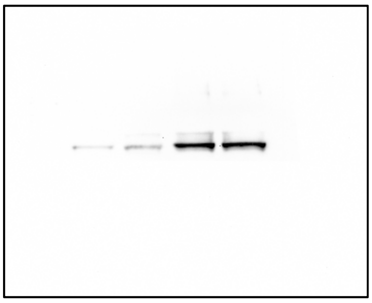

Lane 1_pAKT_ BB342 -10% FBS
Lane 2_pAKT_ BB342 +10% FBS
Lane 3_ pAKT_ BB1866 -10% FBS
Lane 4_ pAKT_ BB1866 +10% FBS

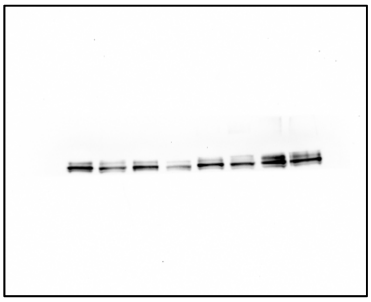

Lane 1_AKT_ BB19 -10% FBS
Lane 2_AKT_ BB19 +10% FBS
Lane 3_ AKT _ BB57 -10% FBS
Lane 4_ AKT _ BB57 +10% FBS
Lane 5_ AKT _ BB1845-10% FBS
Lane 6_ AKT _ BB1845 +10% FBS
Lane 7_ AKT _ BB1865 -10% FBS
Lane 8_ AKT_ BB1865 +10% FBS

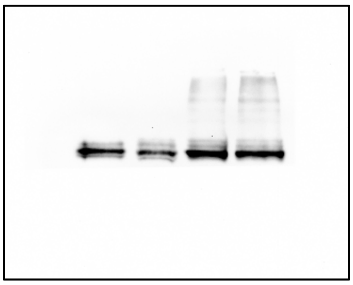

Lane 1_AKT_ BB342 -10% FBS
Lane 2_AKT_ BB342 +10% FBS
Lane 3_ AKT_ BB1866 -10% FBS
Lane 4_ AKT_ BB1866 +10% FBS

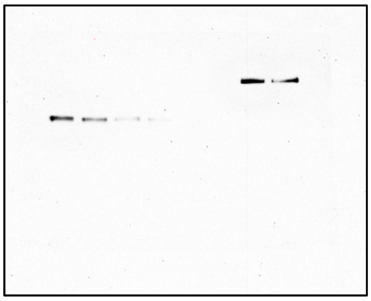

Lane 1_PTEN_ BB19 -10% FBS
Lane 2_PTEN_ BB19 +10% FBS
Lane 3_PTEN _ BB57 -10% FBS
Lane 4_PTEN_ BB57 +10% FBS
Lane 5_ PTEN _ BB1845-10% FBS
Lane 6_ PTEN_ BB1845 +10% FBS
Lane 7_PTEN_ BB1865 -10% FBS
Lane 8_ PTEN_ BB1865 +10% FBS

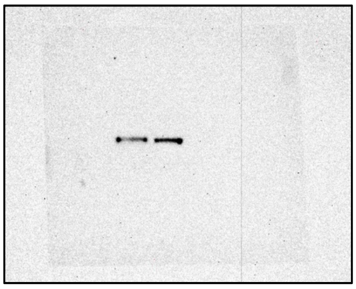

Lane 1_PTEN_ BB342 -10% FBS
Lane 2_PTEN_ BB342 +10% FBS
Lane 3_ PTEN_ BB1866 -10% FBS
Lane 4_ PTEN_ BB1866 +10% FBS

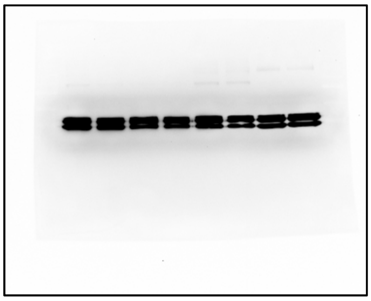

Lane 1_Actin_ BB19 -10% FBS
Lane 2_Actin_ BB19 +10% FBS
Lane 3_Actin _ BB57 -10% FBS
Lane 4_Actin_ BB57 +10% FBS
Lane 5_Actin _ BB1845-10% FBS
Lane 6_Actin_ BB1845 +10% FBS
Lane 7_Actin_ BB1865 -10% FBS
Lane 8_Actin_ BB1865 +10% FBS

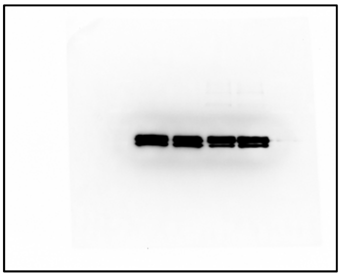

Lane 1_Actin_ BB342 -10% FBS
Lane 2_Actin_ BB342 +10% FBS
Lane 3_ Actin_ BB1866 -10% FBS
Lane 4_ Actin_ BB1866 +10% FBS

b

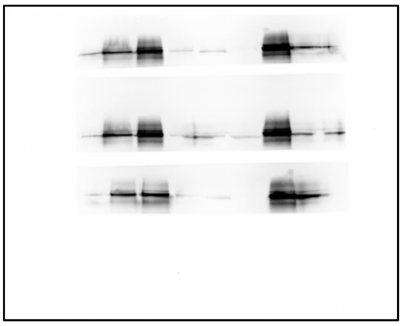

(Triplicate experiment, Lower membrane for figure)
Lane 1_Ecadherin_ BB19
Lane 2_ Ecadherin_ BB57
Lane 3_ Ecadherin_ BB342
Lane 4_ Ecadherin_ BB1845
Lane 5_ Ecadherin_ BB1865
Lane 6_ Ecadherin_ BB1866

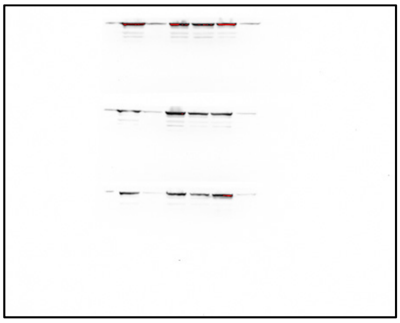

(Triplicate experiment, Lower membrane for figure)
Lane 1_Vimentin_ BB19
Lane 2_ Vimentin_ BB57
Lane 3_ Vimentin_ BB342
Lane 4_ Vimentin_ BB1845
Lane 5_ Vimentin_ BB1865
Lane 6_ Vimentin_ BB1866

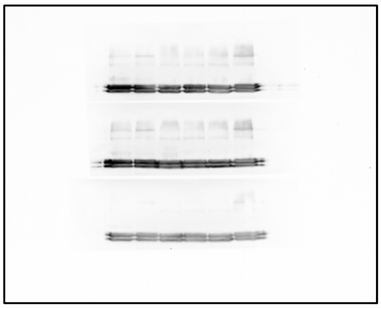

(Triplicate experiment, Lower membrane for figure)
Lane 1_Actin_ BB19
Lane 2_Actin_ BB57
Lane 3_ Actin_ BB342
Lane 4_ Actin_ BB1845
Lane 5_ Actin_ BB1865
Lane 6_ Actin_ BB1866

Figure S5. (a) Original, uncropped Western blot membrane in Figure 2C; (b) Original, uncropped Western blot membrane in Figure 3B.
