# Supplementary material for: Development of Novel Murine BRAFV600E-Driven Papillary Thyroid Cancer Cell Lines for Modeling of Disease Progression and Preclinical Evaluation of Therapeutics
Source: Cancers (Basel). 2023 Jan 31;15(3):879. doi: 10.3390/cancers15030879 (PMC9913801; doi:10.3390/cancers15030879)
Supplement: Supplementary file 1 [file cancers-15-00879-s001.zip › cancers-1981262 -Figures S1-S4.pdf]

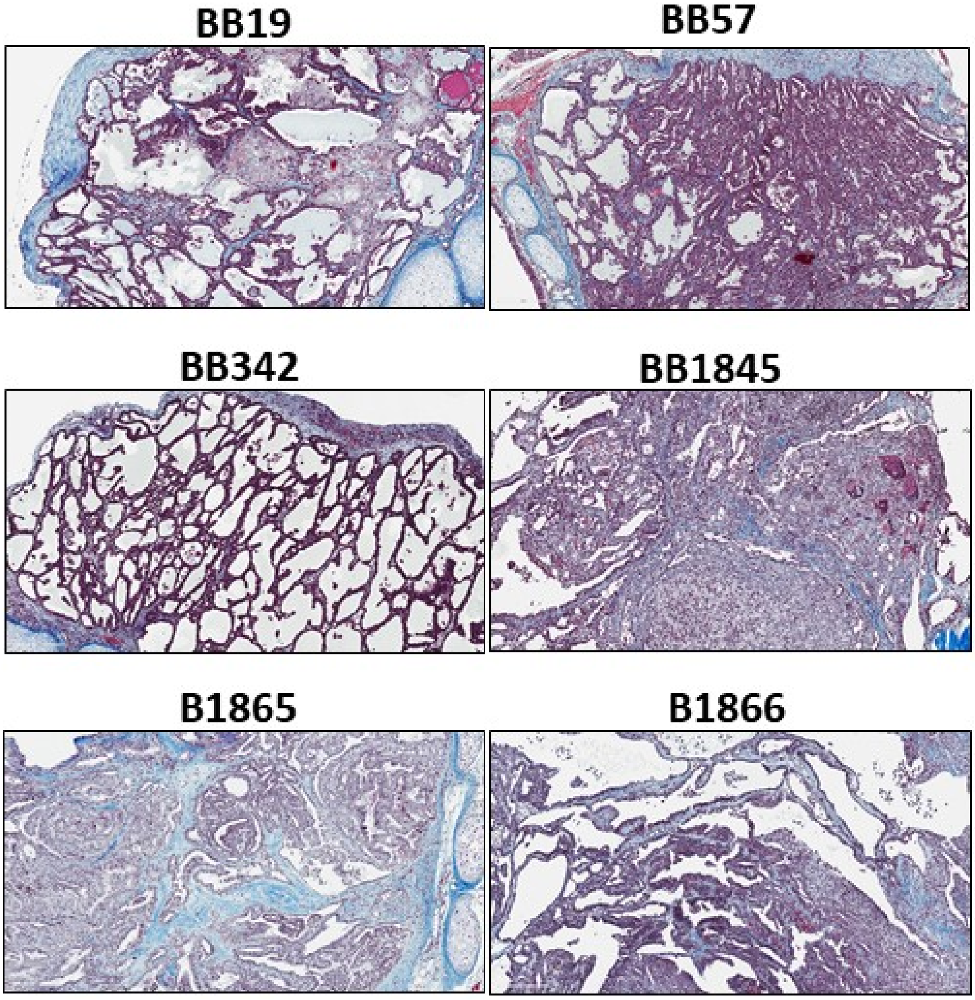

Figure S1. Trichrome of original de novo tumors. *BrafV600E*$^{+/-}$*/Pten*$^{-/-}$*/TPO-Cre de novo* tumors contain deposits of collagen, as shown in representative trichrome stained images. Collagen is blue, nuclei are black, and muscle tissue, erythrocytes and cytoplasm are red to pink. Collagen tracts of varying sizes can be observed in all the tumors. The three more advanced tumors (BB1845, B1865 and B1866) exhibit larger, more well defined collagen tracts than the three more well differentiated tumors (BB19, BB57, and BB342).

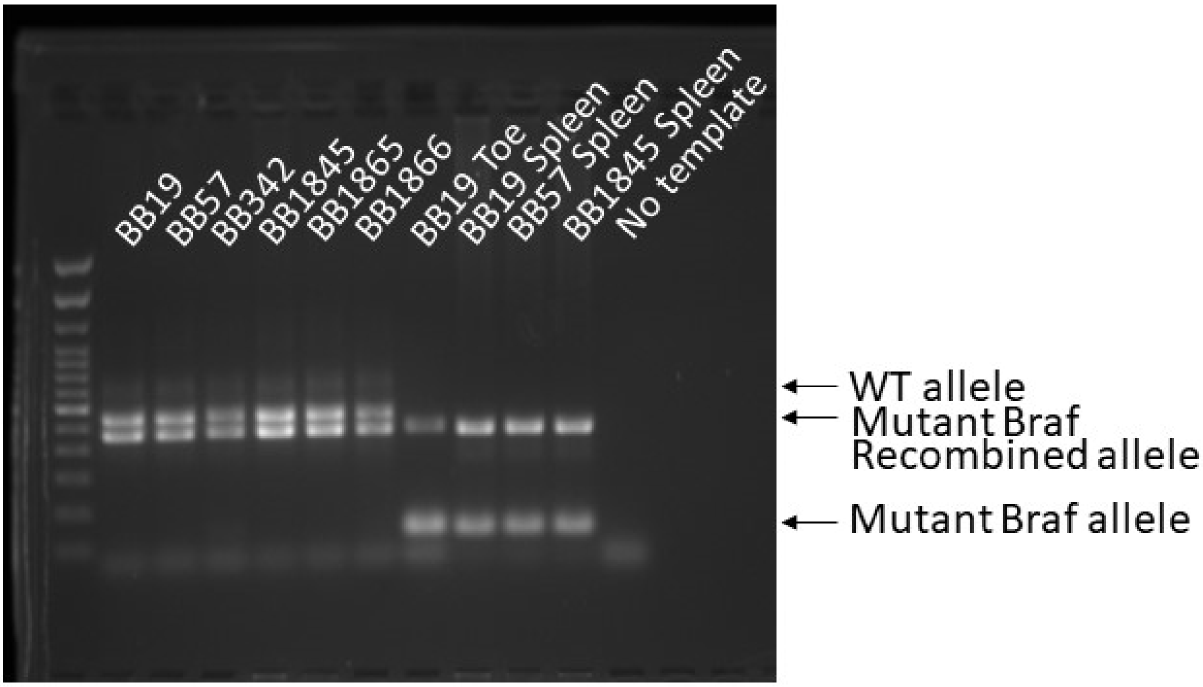

Figure S2. Braf Recombined DNA gels on cell lines and non-target tissues. Polymerase chain reaction (PCR) analysis of DNA from the tumor cell lines and control tissue to detect presence of WT, unrecombined allele and recombined allele of *Braf*. First lane is a 100 base pair ladder. The WT, mutant unrecombined Braf and mutant recombined *Braf* allele bands are indicated. Non-thyroid control tissue and no template control are included as indicated.

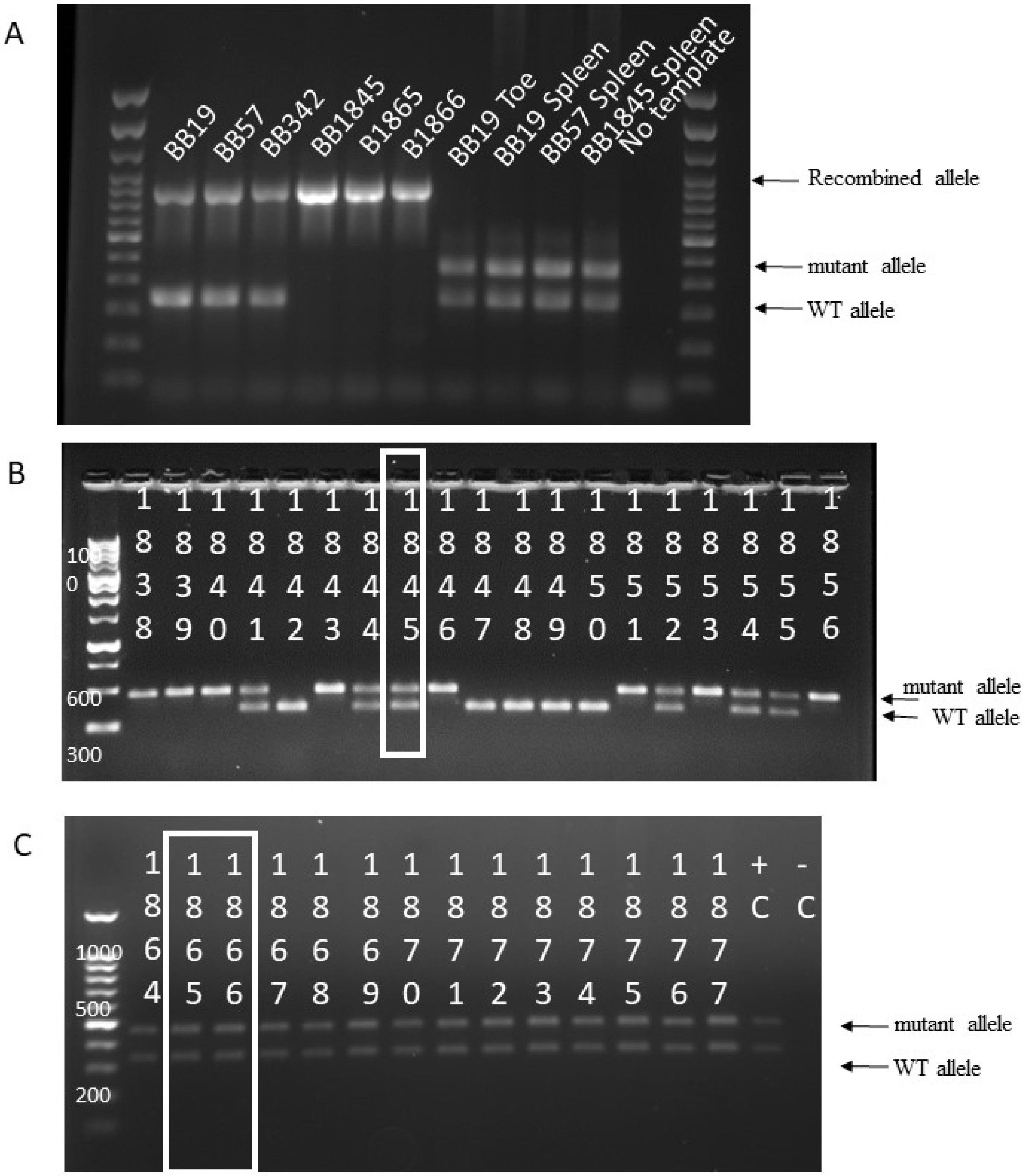

Figure S3. Pten Recombined DNA gel on cell lines and non-target tissues and original Pten Genotyping gels for BB1845, B1865, and B1866. (**A**) Polymerase Chain reaction (PCR) analysis of PTEN recombination in tumor cell lines and non-thyroid control tissue. Note the presence of the PTEN recombined allele only in the tumor cell lines but not non-thyroid control tissue. The WT allele is only present in tumor cell lines BB19, BB57 and BB342 as well as all control non-thyroid tissue. Control non-thyroid tissue also has the unrecombined PTEN allele (**B**) Original Pten Genotyping gel from tail DNA of mouse from which tumor BB1845 was collected indicating that the animal was indeed a $PTEN^{Het}$, but has subsequently lost the WT allele in the tumor cell line. (**C**) Original Pten Genotyping gel from tail DNA of mice B1865 and B1866 from which tumor cell lines B1865 and B1866 were collected indicating that the animals were indeed a $PTEN^{Het}$, but have subsequently lost the WT allele in the tumor cell line.

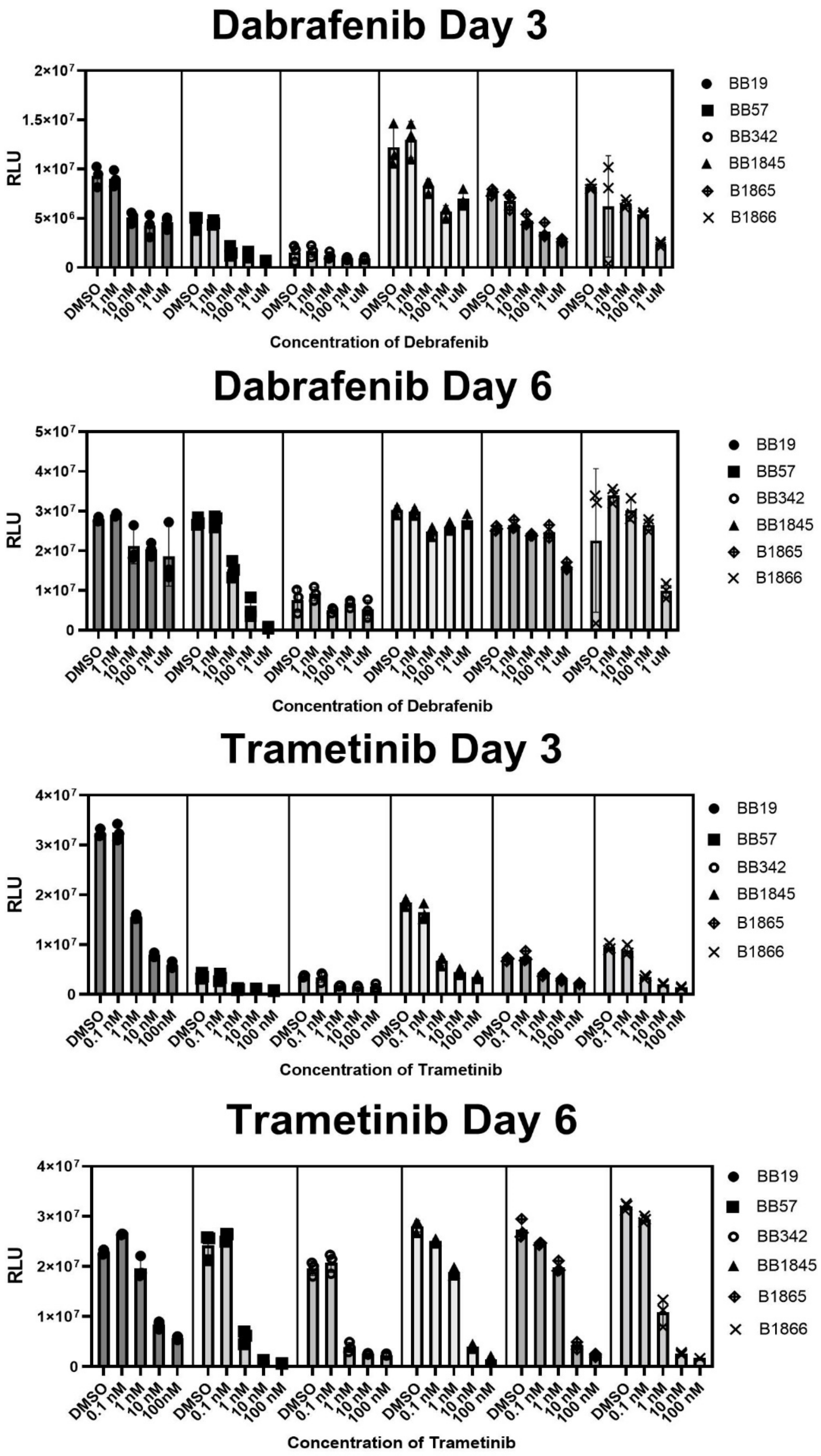

Figure S4. Dose-dependent growth-suppressive effects of targeted BRAF and MEK1/2 inhibition in *BrafV600E+/−/Pten+/−/TPO-Cre* cell lines. (A) Summary plot showing dose-dependent viability of novel cell lines in 2D culture following treatment with the BRAF inhibitor Dabrafenib, MEK1/2 inhibitor Trametinib or DMSO vehicle compared to control cells grown in standard culture medium. Cells were treated with inhibitors for 3 or 6 days prior to analysis using CellTiter Glo Luminescent Cell Viability Assay.
